# Identification of hub genes in bladder transitional cell carcinoma through ceRNA network construction integrated with gene network analysis

**DOI:** 10.1111/jcmm.17979

**Published:** 2023-10-05

**Authors:** Hai Wang, Lei Gao, Yin Chen, Lei Zhang, Yu Bai, Cuiping Zhao, Lifeng Zhang, Li Zuo, Heyun Sun

**Affiliations:** ^1^ Department of Oncology The Affiliated Jintan Hospital of Jiangsu University Changzhou China; ^2^ Department of Urology Changzhou Second People's Hospital Changzhou China; ^3^ Department of Geriatrics Changzhou Second People's Hospital Changzhou China

**Keywords:** bladder cancer, ceRNA, network, transitional cell carcinoma, WGCNA

## Abstract

Bladder transitional cell carcinoma (BTCC) forms more than 90% of bladder cancer cases. It brings challenges to the early diagnosis and therapy of BTCC, due to lack of efficient screening biomarkers. We used weighted gene co‐expression network analysis (WGCNA) combined competing endogenous RNA (ceRNA) network construction depending on TCGA datasets to investigate potential hub genes and regulatory pathways associated with occurrence and progression of BTCC. We further used real‐time polymerase chain reaction (RT‐PCR) to validate the relative expression genes correlated with BTCC. By WGCNA, the gene co‐expression module with 11 genes was found corelated with BTCC tumour stage and prognosis after survival analyses. Ultimately, we put 100 highly stage‐related genes into the above constructed ceRNA network and then constructed another new network. Among them, all elements in AC112721.1/LINC00473/AC128709.1‐hsa‐mir‐195‐RECK and LINC00460‐hsa‐mir‐429‐ZFPM2 axes were simultaneously corelated with overall survival. RT‐PCR showed that AKAP12 was downregulated in tumour tissues. The hub genes screened out in the present study may provide ideals for further treatment on BTCC.

## 1 INTRODUCTION

Bladder transitional cell carcinoma (BTCC) is still the most common urologic cancers worldwide. In the United States, approximately 80,470 new patients and 17,670 cancer‐related deaths were expected in 2019.^1^ The morbidity of BTCC has constantly elevated since the 1990s, although the medical techniques, the diagnostics and treatments kept on advancing.^2^ BTCC is also called bladder urothelial carcinoma and forms more than 90% of bladder cancer cases.^3^ Despite the majority of BTCC are non‐invasive, about 25% tend to metastasize and progress to muscle‐invasive disease eventually.^4^ Early stage BTCC patients possess a preferable 5‐year survival rate, while muscle‐invasive bladder cancer and metastasized tumour account for the main cause of death.^5^ Due to absence of efficient screening biomarkers, it brings challenges to the early diagnosis and therapy of BTCC.^6^ Hence, it is promising to investigate underlying molecular mechanism and identify more effective biomarkers for early diagnosis, prognostic analysis and searching appropriate therapeutic targets for patients with BTCC.

Cancer genomics has achieved constant development in recent years due to rapid evolution of microarray and high‐throughput sequencing techniques. By integrated bioinformatic methods, various cancer‐associated molecular biomarkers, ranging from coding genes to noncoding genes, have been identified in biologic and clinical aspects, and provide ideas and direction for experiments.^7^, ^8^ Evidence has been established that many protein‐coding RNAs and noncoding RNAs can participate in the occurrence and development of many malignancies, while the underlying mechanisms in BTCC were obscure and remain to be explored.

Competitive endogenous RNA (ceRNA) network was used to better clarify the mutual regulatory mechanisms of those protein‐coding RNAs and noncoding RNAs, which can participate in biological process of cancer.^9^ Long‐noncoding RNAs (lncRNAs) can act as ceRNA, which may be associated with mRNAs and has gained widespread recognition.^10^, ^11^ Previously, some researchers revealed that lncRNAATB can competitively bind to the miR‐200 family and subsequently promote tumour invasion.^12^ Thus, it will be helpful to seek out key lncRNA, miRNA and miRNA in BTCC by construction of ceRNA network. Weighted gene co‐expression network analysis (WGCNA) was firstly designed as a method which could relate gene expression to phenotypic traits by building co‐expression modules, and has widely been used to explore molecular biomarkers for several cancers.^13^, ^14^, ^15^ Herein, we attended to find out potential key gene regulatory pathways associated with tumour stage of BTCC by WGCNA combined ceRNA network construction depending on RNA expression profile in TCGA database.

## 2 MATERIALS AND METHODS

### 2.1 Patients and samples

Eight patients who were pathologically diagnosed with bladder cancer were used in present study. The study followed the reporting recommendations for studies on prognostic tumour markers (REMARK). The study was also approved by the institutional ethical review boards of the hospitals, and written informed consent was obtained from all bladder cancer patients. The clinicopathological features of all the bladder cancer patients are listed in Table S1.

### 2.2 Data collection

We retrieved the gene expression data and clinic traits of patients with bladder transitional cell carcinoma using The Cancer Genome Atlas (TCGA) database (https://cancergenome.nih.gov/). A total of 411 tumour patients and 19 controls containing the mRNA and lncRNA expression data were included. Additionally, the miRNA expression data was comprised of 415 tumour participants and 19 controls. Moreover, the clinical information was from 405 patients.

### 2.3 Screening differentially expressed genes

We used the ‘edgeR’ R package to investigate the differentially expressed mRNAs, lncRNAs and miRNAs (represents for DEmRNAs, DElncRNAs and DEmiRNAs, respectively) between BTCC samples and normal counterparts.^16^ The threshold should be in accord with both |log_2_FoldChange|more than 2 and *p*_adj_ value <0.05.

### 2.4 CeRNA network analysis

We used the miRcode to evaluate the interactions of lncRNA‐miRNA.^17^ Target mRNAs of miRNAs should simultaneously meet with three databases (miRTarBase, miRDB and TargetScan) to construct the network.^18^, ^19^, ^20^ Finally, a Cytoscape 3.6.1 software was adopted to visualize the lncRNA‐miRNA‐mRNA network.

### 2.5 Co‐expression and module analysis

Totally, 403 patients with sufficient clinical information of tumour stage, race, years of diagnose and survival status were involved in the process of WGCNA with their unique gene expression profiles. The DEmRNAs screened in above steps were chosen to build co‐expression network. The power *β* was calculated according to a scale‐free topology criterion.^21^ Afterwards, Gene modules were conducted using hierarchical clustering dendrogram. The topological overlap matrix (TOM) was used for visualization. Finally, we assessed the correlations between modules and clinical characteristics and identified the positive genes. The clinical traits of enrolled participants were provided in Table S2.

### 2.6 Gene ontology (GO) analysis

We carried out the GO and Kyoto Encyclopedia of Genes and Genomes (KEGG) analysis based on the David 6.8 program (http://david.abcc.ncifcrf.gov/).

### 2.7 Real‐time polymerase chain reaction (RT‐PCR)

Real‐time PCR assays were carried out based on the following protocol. Briefly, total RNA from human tissues was extracted using TRIzol Reagent (15,596,018, Gibco). cDNA was synthesized using the PrimeScript One Step RT Reagent Kit (RR064A, TaKaRa) following the manufacturer's instructions. Quantitative RT‐PCR was performed using a StepOnePlus Real‐Time PCR System (Applied Biosystems) and SYBR Green Real‐Time PCR Master Mix (QPK201, Toyobo). All results were normalized to the expression of glyceraldehyde 3‐phosphate dehydrogenase (GAPDH). The fold change relative to the mean value was determined by the $2^{-\Delta \Delta C_{t}}$ method. The primer sequences of validated genes were presented as follows:

ZFPM2, 5′‐GCAAGGAGTGGAAGACAACAAA‐3′ (forward) and 5′‐ATACCAGATGCCACAGGACTTG‐3′ (reverse), 292 bp.
AKAP12, 5′‐GCAAAGAGGAAGGAGAAGAGAAAC‐3′ (forward), and 5′‐ACTTCATCCTCCTTCGGCTTC‐3′ (reverse), 169 bp.
ZEB1, 5′‐GGCATACACCTACTCAACTACGG‐3′ (forward) and 5′‐CATTCCATTTTCTGTCTTCCGCA‐3′ (reverse), 187 bp.
RECK, 5′‐AATGAGGAACCCAACGGATAGT‐3′ (forward) and 5′‐TTTCCGTTTTCTTGGACATCAGG‐3′ (reverse), 101 bp.
The β‐actin used as normalization: 5′‐CACCCAGCACAATGAAGATCAAGAT‐3′ (forward) and 5′‐ CCAGTTTTTAAATCCTGAGTCAAGC‐3′ (reverse).

### 2.8 Survival evaluation

Survival curves of screened mRNAs, miRNAs and lncRNAs were performed by ‘survival’ R package with a Kaplan–Meier univariate survival method.^22^

## 3 RESULTS

### 3.1 Identification of DEmRNAs, DElncRNAs and miRNA

**FIGURE 1:**
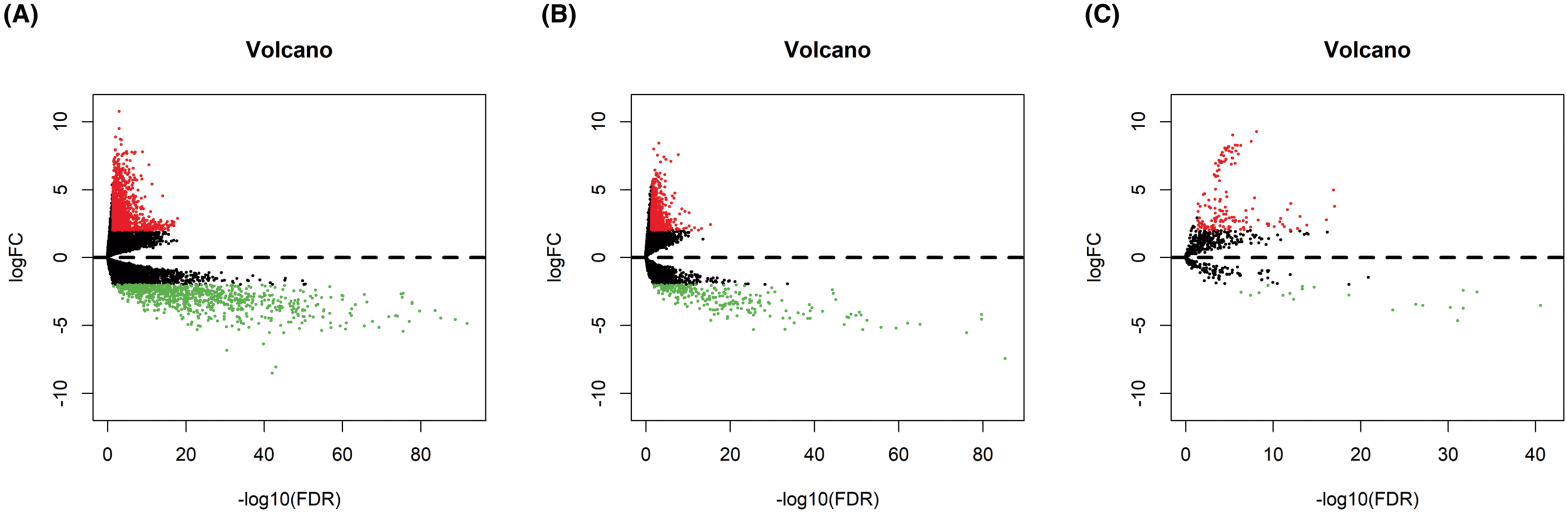
Volcano plots of these different expressed mRNAs (A), lncRNAs (B) and miRNAs (C).

The mRNA, lncRNA and miRNA expression profiles of patients with BTCC were evaluated according to the TCGA database. Totally, 2114 DEmRNAs, 918 DElncRNAs and 171 DEmiRNAs were screened out with edgeR package. The upregulated RNAs included 1268 mRNAs, 659 lncRNAs and 150 miRNAs, while 846 mRNAs, 259 lncRNAs and 21 miRNAs were downregulated. Figure 1 was the volcano plots of these different expressed RNAs.

### 3.2 CeRNA network analysis

**FIGURE 2:**
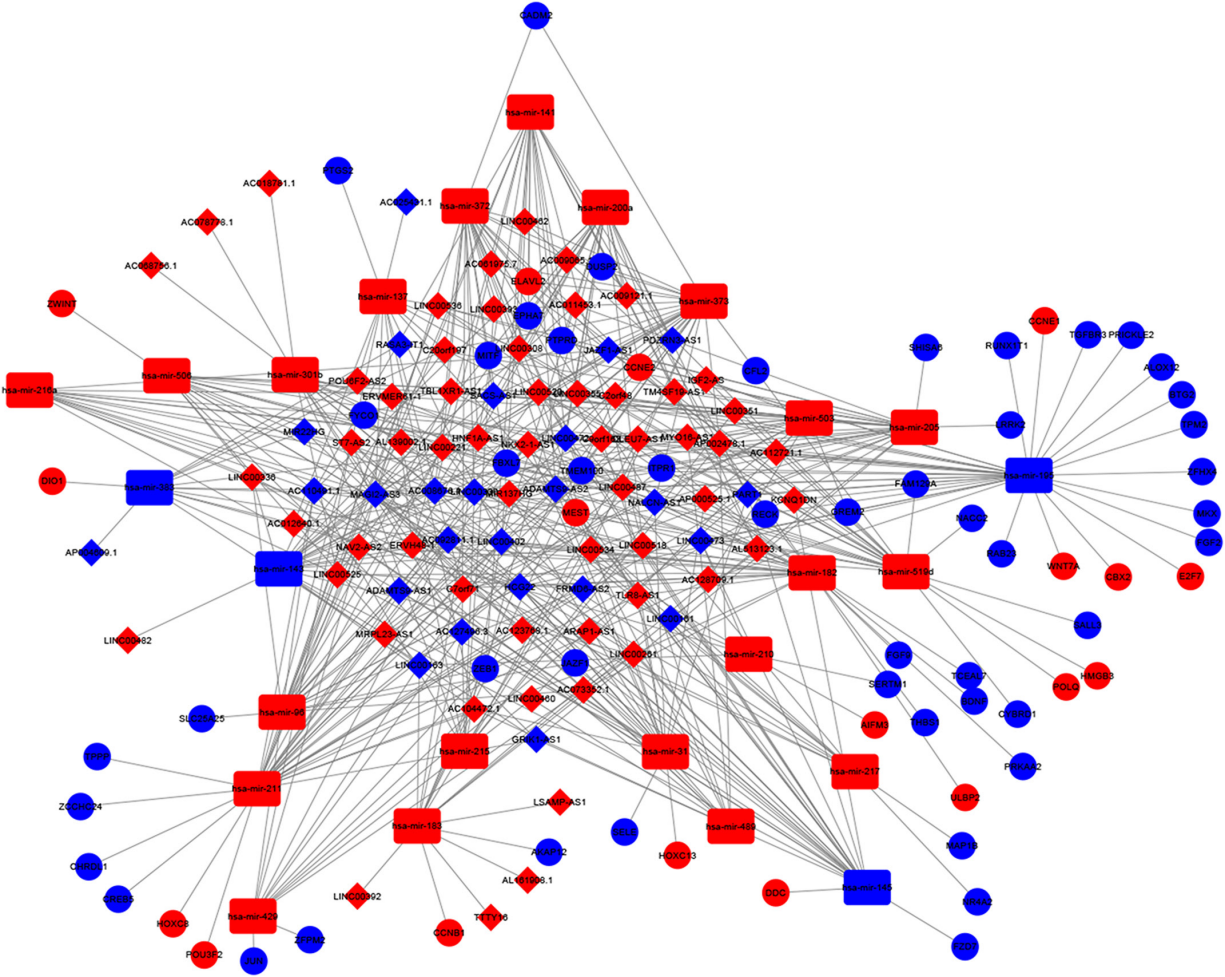
CeRNA network with 83 lncRNAs, 25 miRNAs and 67 mRNAs.

Firstly, lncRNA–miRNA pairs were retrieved from miRcode. We used miRTarBase, miRDB and TargetScan to predict the target mRNAs of these miRNAs. Only those simultaneously meet with the three databases was selected. Ultimately, the lncRNA–miRNA–mRNA network was conducted with 83 lncRNAs, 25 miRNAs and 67 mRNAs, and visualized by Cytoscape (Figure 2).

### 3.3 GO pathway analysis in ceRNA network

**FIGURE 3:**
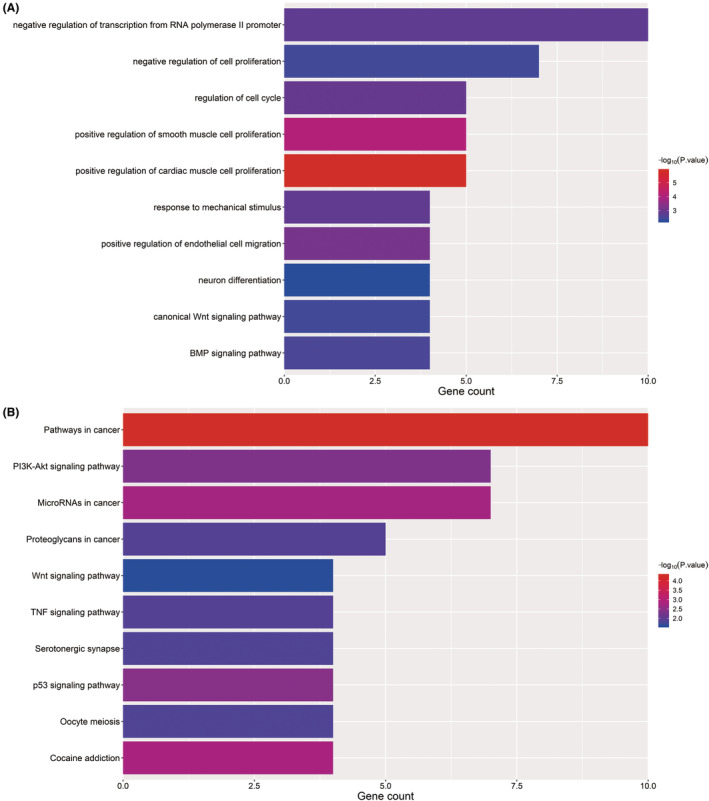
Go terms for biological process (A) and KEGG pathway analyses (B) of the 67 mRNAs.

GO and KEGG pathway enrichment analyses of the 67 genes in ceRNA network were carried out according to the Database for Annotation, Visualization and Integrated Discovery (DAVID, http://david.abcc.ncifcrf.gov/). Negative regulation of cell proliferation and transcription from RNA polymerase II promoter were the top GO terms for biological processes. The main KEGG pathways of them focused on cancer‐related pathways, MicroRNAs and PI3K‐Akt signalling pathway, which revealed these pathways might be related to the BTCC occurrence. The most positive GO terms of biological process and enriched KEGG pathways were shown in Figure 3.

### 3.4 Weighted gene co‐expression network analysis (WGCNA)

**FIGURE 4:**
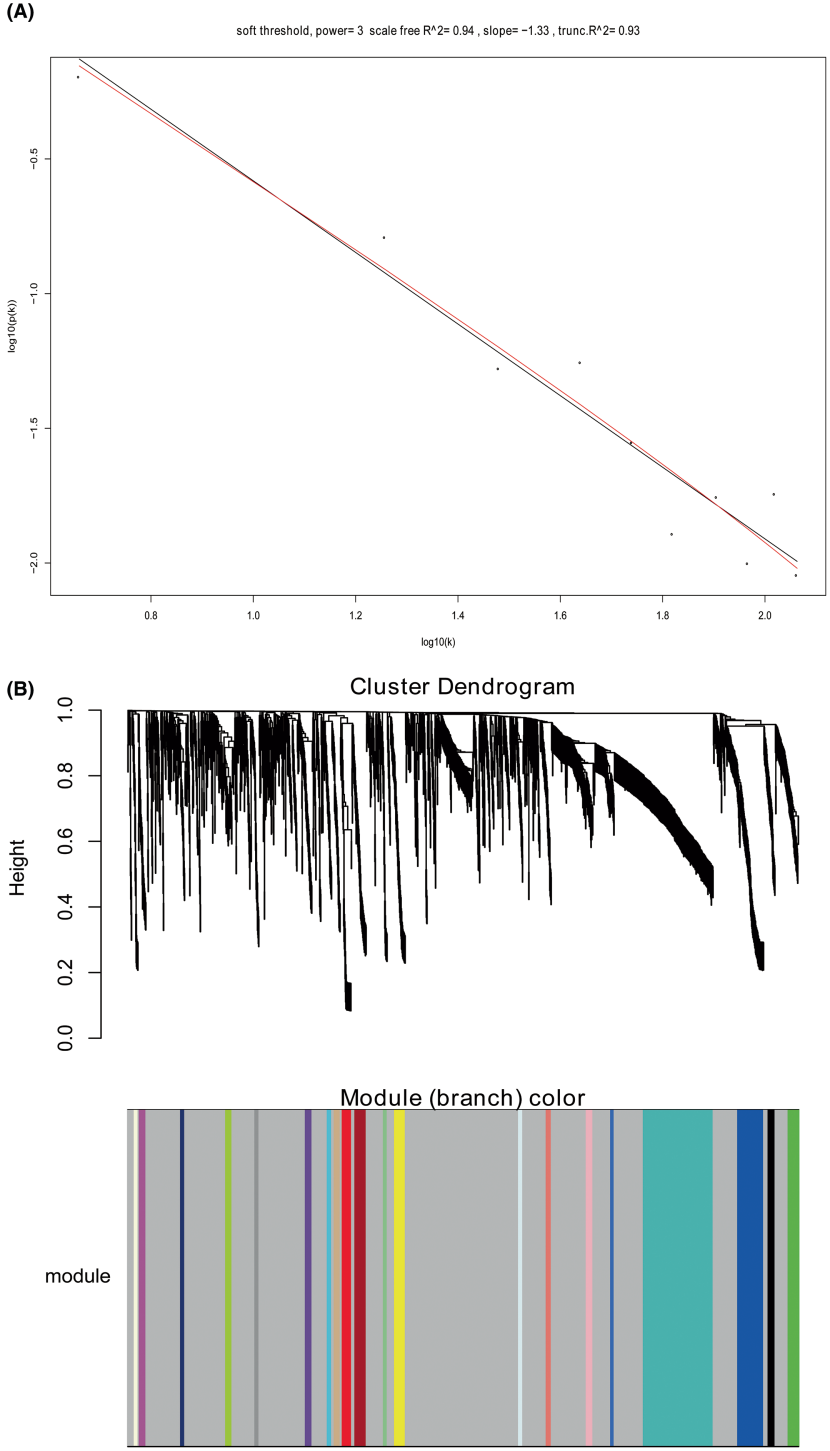
Gene co‐expression modules construction. (A): Soft threshold test with a *β* = 3; (B) Dendrogram of all differentially expressed gene co‐expression modules.

**FIGURE 5:**
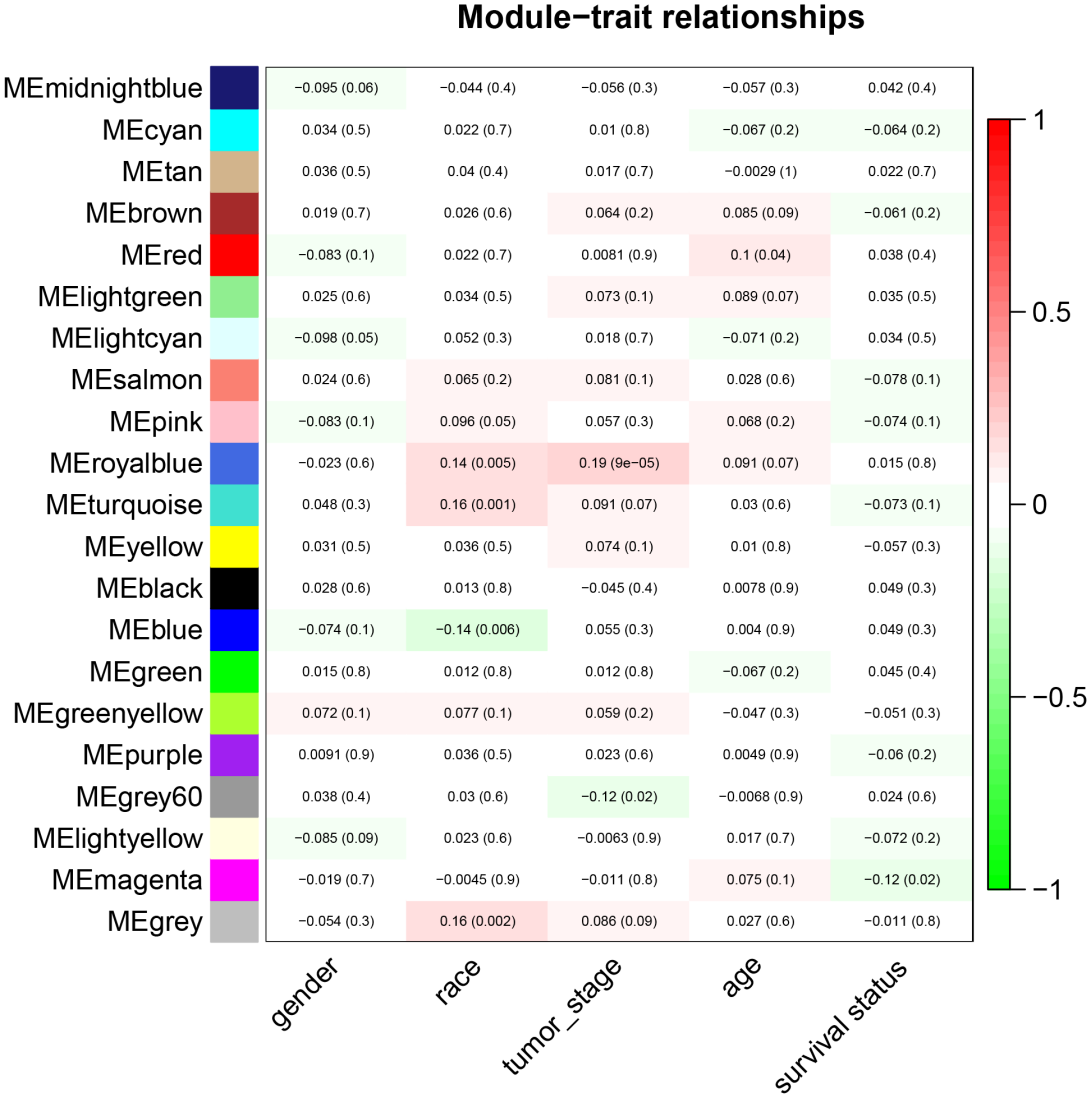
Heatmap of the correlation between module genes and clinical traits of BTCC.

**FIGURE 6:**
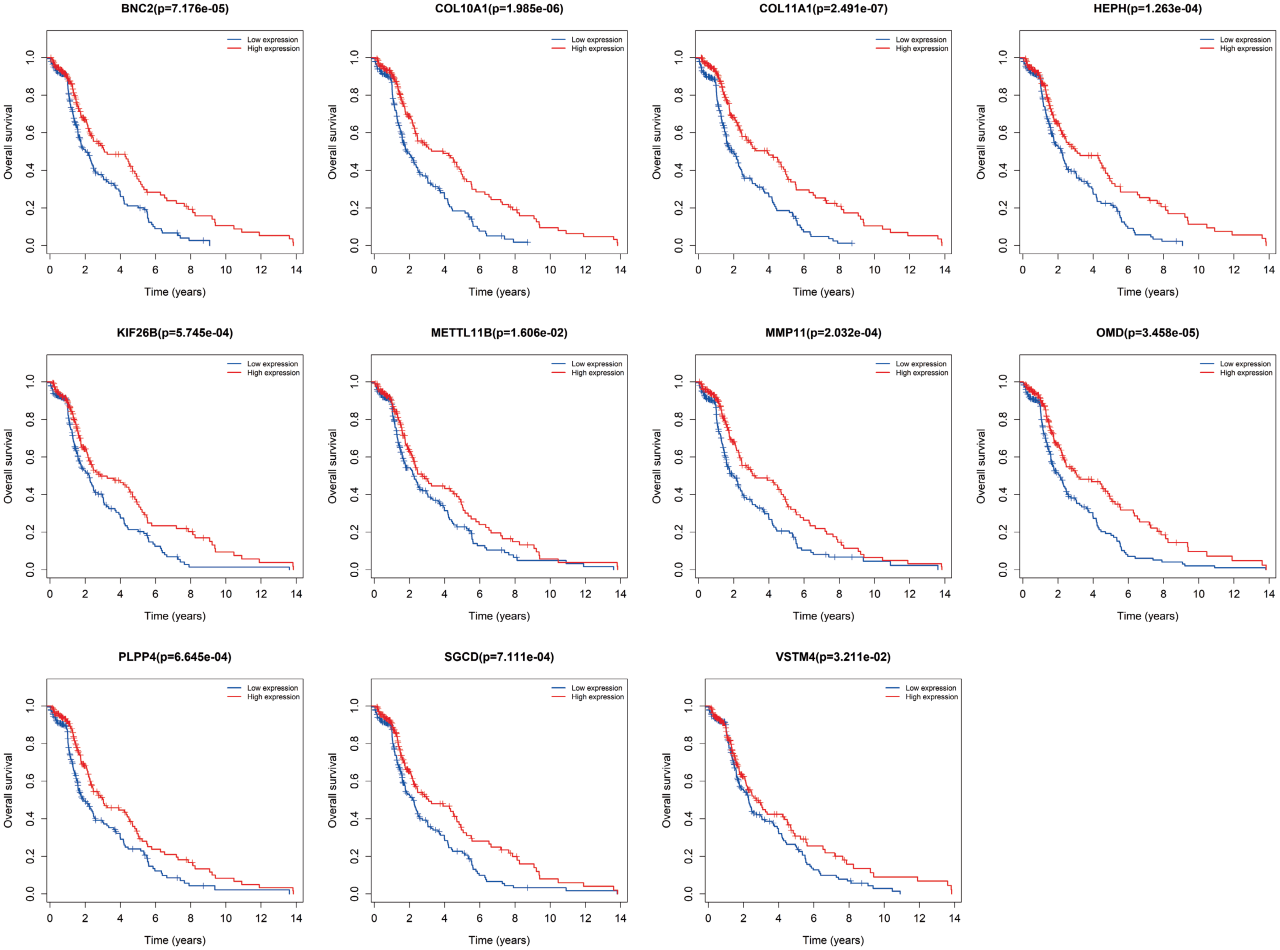
Survival curves of 11 genes in the royal blue module.

WGCNA algorithm was adopted to structure the co‐expression modules with 2114 DEmRNAs. The power of *β* = 3 was the soft‐thresholding after test to perform gene modules analysis (Figure 4A). We structured 21 gene modules (Figure 4B) based on clinical traits, including gender, race, tumour stage, age and survival status (Figure 5). We found the royal blue module with 11 genes was most significantly associated with tumour stage. We next conducted survival analyses of these 11 genes (BNC2, COL10A1, COL11A1, HEPH, KIF26B, METTL11B, MMP11, OMD, PLPP4, SGCD and VSTM4) and drew Kaplan–Meier survival curves (Figure 6). As a result, they all corelated with prognosis, which revealed they might be hub genes of BTCC and worth to further study of their specific roles.

### 3.5 Combination of ceRNA network and validation of RT‐PCR

**FIGURE 7:**
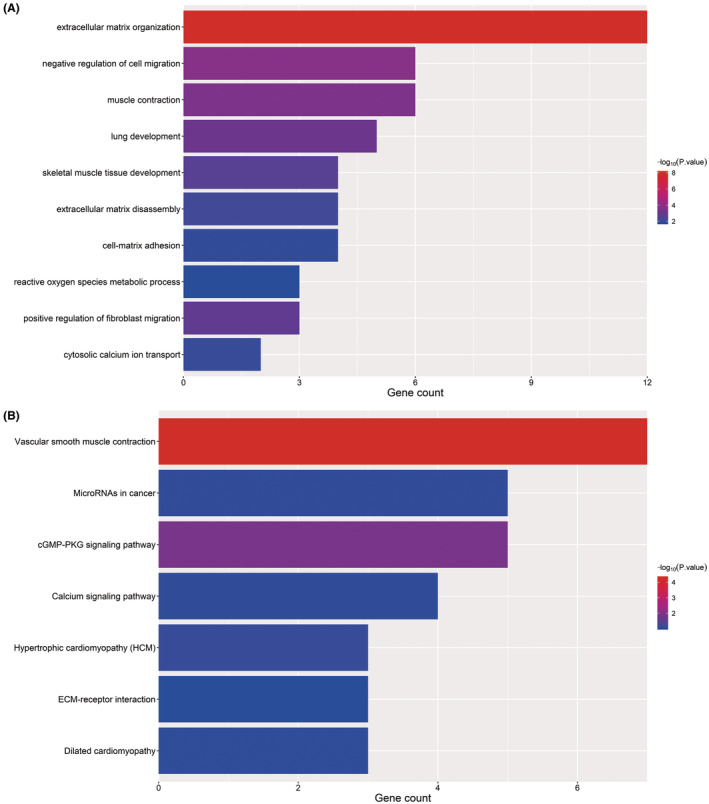
Go terms for biological process (A) and KEGG pathway analyses (B) of the 101 highly stage‐corelated genes.

**FIGURE 8:**
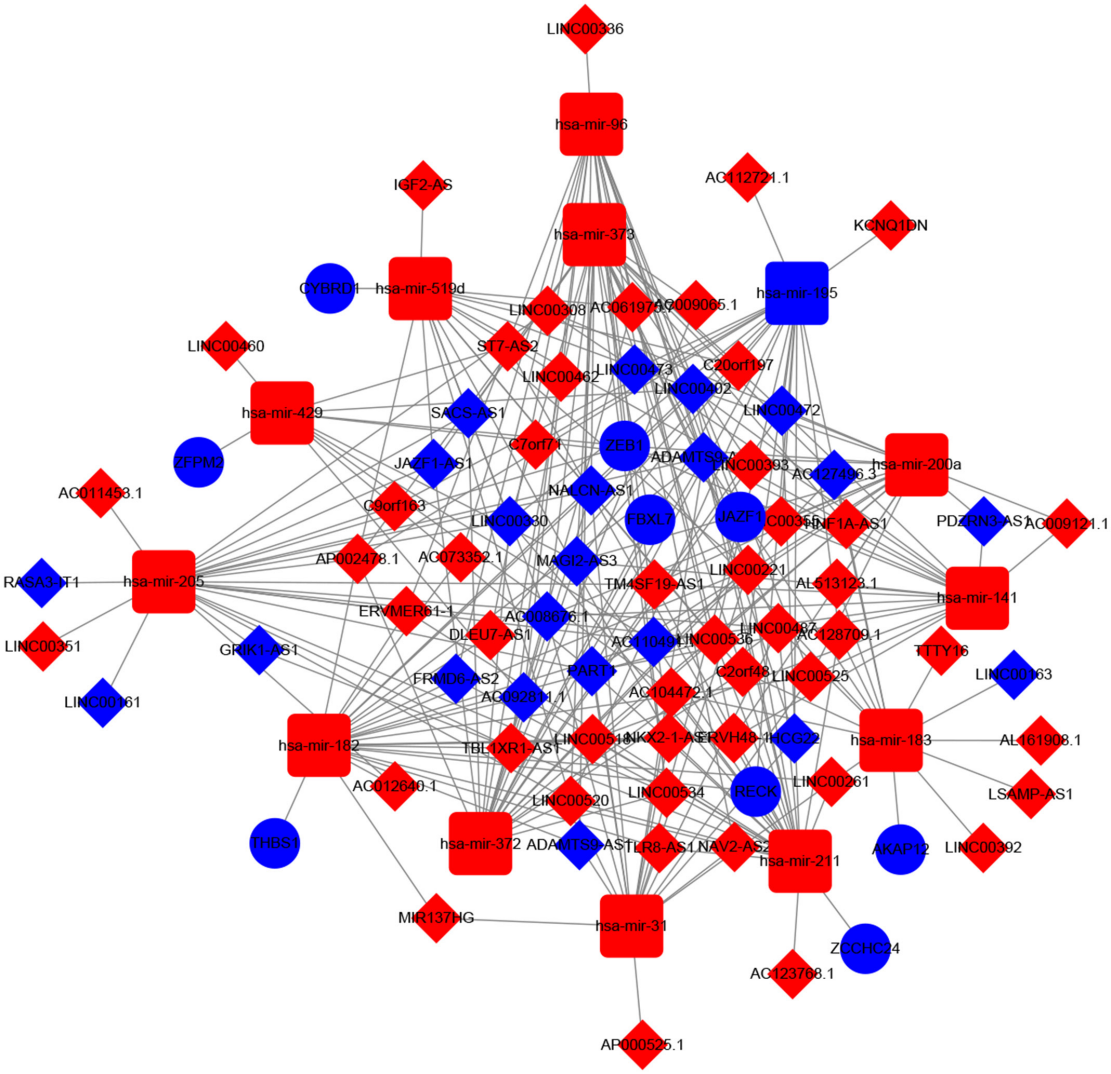
CeRNA network with 9 mRNAs, 13 miRNAs and 70 lncRNAs when combined with WGCNA.

**FIGURE 9:**
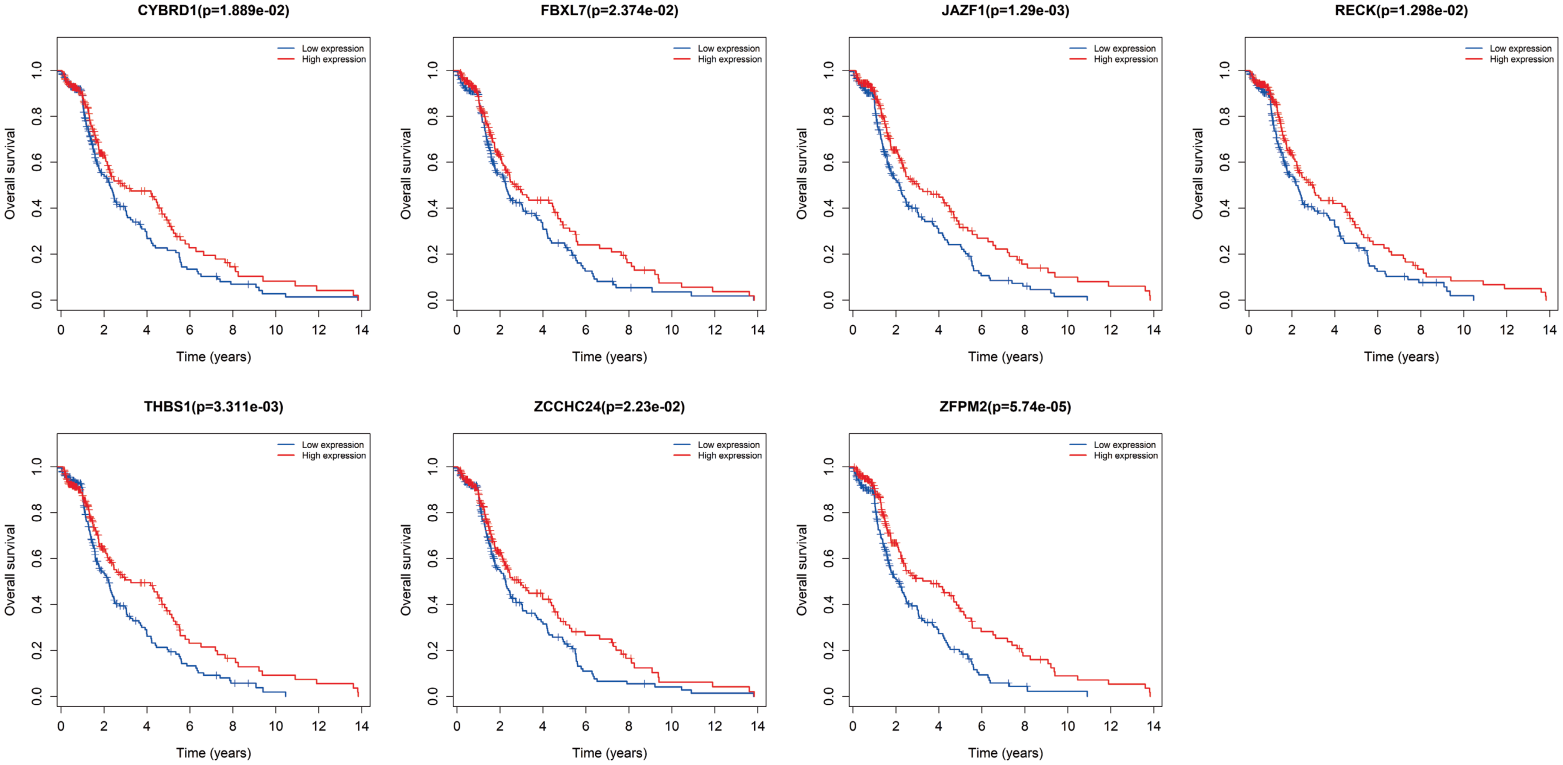
Survival curves of mRNAs in network with a *p* < 0.05.

**FIGURE 10:**
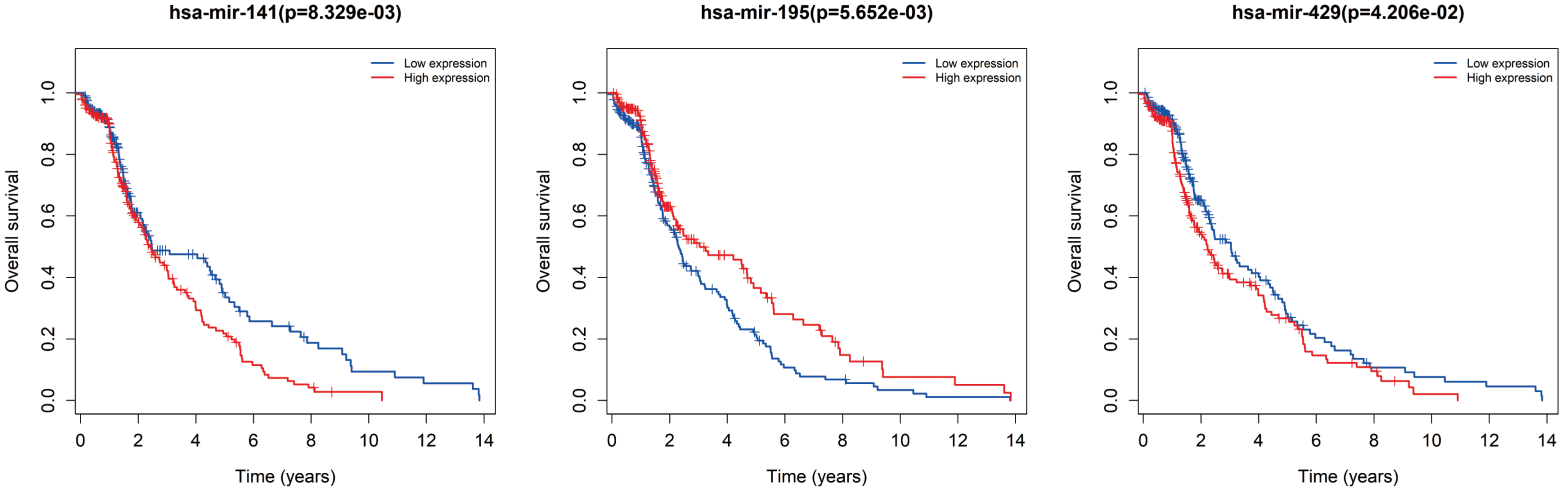
Survival curves of miRNAs in network with a *p* < 0.05.

**FIGURE 11:**
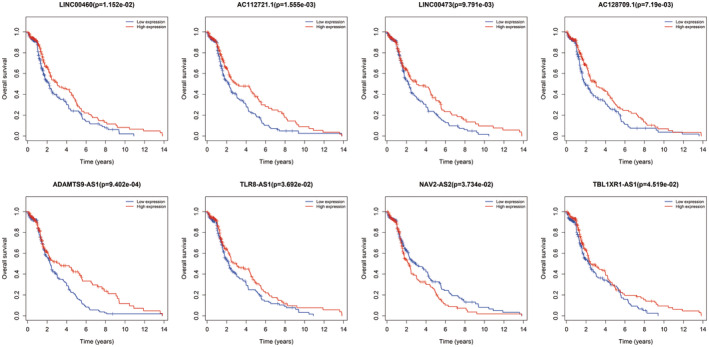
Survival curves of lncRNAs in network with a *p* < 0.05.

**FIGURE 12:**
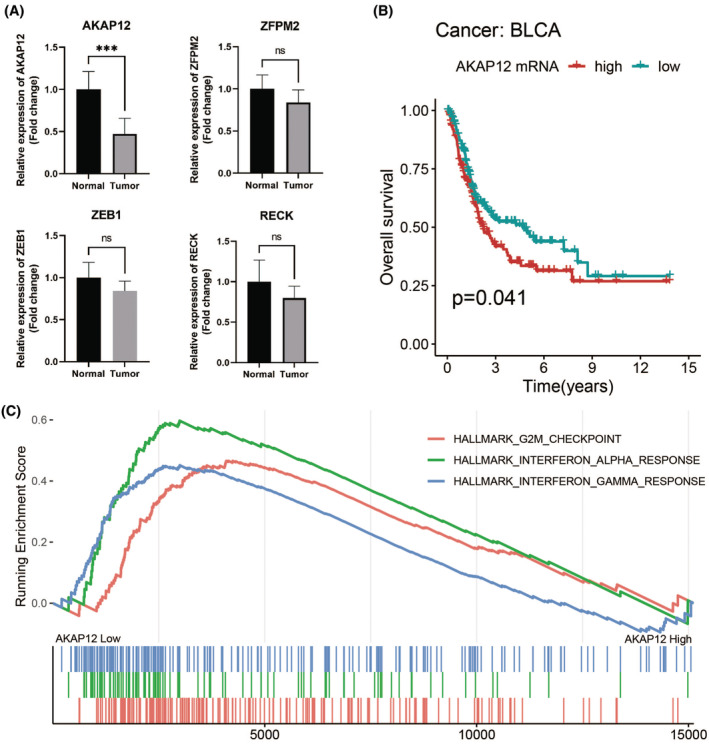
(A)The relative mRNA expression of various genes. (B) The survival curve of BTCC patients in terms of overall survival. (C) Gene set enrichment analysis of AKAP12 in BTCC based on the TCGA cohort.

Due to no common genes between ceRNA network and the most tumour stage‐related module, we chose the significantly corelated gene with tumour stage in all modules with a threshold *p* < 0.01 and Cor >0.1, which contained 100 genes (Table S3). Figure 7 showed the main GO terms of biological process and KEGG pathways of them. Finally, we find nine genes both in ceRNA network and corelated with BTCC stage, including ZFPM2, AKAP12, ZEB1, RECK, THBS1, CYBRD1, JAZF1, FBXL7 and ZCCHC24. New ceRNA network were constructed surrounding these 9 mRNAs, as well as their upstream 13 miRNAs and 70 lncRNAs (Figure 8). In addition, we performed survival analyses with all RNAs in the new ceRNA network. Ultimately, seven mRNAs, three miRNAs and eight lncRNAs were revealed to show significance with the prognosis of tumour patients (Figures 9, 10, 11). Moreover, the pairs of lncRNA‐miRNA‐mRNA which were all significant simultaneously were screened out (AC112721.1/LINC00473/AC128709.1‐hsa‐mir‐195‐RECK and LINC00460‐hsa‐mir‐429‐ZFPM2). They might be potential key regulatory pathways of the progression of BTCC and could guide the experimental validation later on. Moreover, we conducted the RT‐PCR to validate the relative expression of four genes (ZFPM2, AKAP12, ZEB1 and RECK). Our study showed that AKAP12 was downregulated in cancer tissues, as compared to control (*p* < 0.05), which further confirmed the conclusion of the current analysis. No significant difference was revealed for ZFPM2, ZEB1 and RECK (*p* > 0.05, Figure 12A). Moreover, we evaluated the prognosis of AKAP12 in bladder cancer (Figure 12B). The mean expression of AKAP12 was set as the cut‐off value. As visualized that the survival rate of patients with high expression of AKAP12 was lower than those with low expression of AKAP12. Besides, the function of AKAP12 was evaluated via GSEA based on the data derived from the BLCA‐TCGA cohort. Results showed that AKAP12 might be involved in cell cycle and immune regulation in bladder cancer progression for the observed enrichment of G2M checkpoint and interferon α/γ response pathway in low AKAP12 expression group (Figure 12C).

## 4 DISCUSSION

As estimated in the GLOBOCAN 2018 database, there would be 549,393 new BTCC patients and 199,922 deaths among 185 countries worldwide in 2018.^23^ BTCC is the most frequent pathological subtype of bladder cancer and has seriously threatened the human health due to its poor survival expectation.^24^, ^25^ Up to now, insufficient biomarkers have been found dominant in the occurrence and progression of BTCC. One of the most significant reasons may be the lack of a deep understanding of the molecular mechanisms in this process.

Recently, the role of noncoding RNAs, which includes lncRNA, miRNA circular RNA and so on, has been validated in various process in BTCC of their pre or post transcriptional regulation on protein‐coding mRNAs, and was expected to be superior biomarkers for tumour advance and prognosis.^26^, ^27^, ^28^ For example, Zhan et al.^29^ revealed that lncRNA PANDAR was correlated with poor prognosis and tumorigenesis in bladder urothelial. What's more, the proposition and validation of ceRNA hypothesis further provided clues for potential mechanisms in BTCC.^30^, ^31^ In addition, as another pop method for bioinformatics, WGCNA was characterized by its distinct advantages in identification co‐expression gene modules relevant to clinical features.^32^ Hence, we aimed to combined the two methods at the same time in the present study to minimizing the range of hub genes in BTCC.

Firstly, we acquired 2114 DEmRNAs, 918 DElncRNAs and 171 DEmiRNAs in virtue of the huge samples of BTCC in TCGA database. Referred to miRcode, miRTarBase, miRDB and TargetScan database, we built a ceRNA network combined with 83 lncRNAs, 25 miRNAs and 67 mRNAs. Functional enrichment analysis showed the top GO terms of 67 genes were negative regulation of cell proliferation and transcription from RNA polymerase II promoter. The main KEGG pathways focused on cancer‐related pathways, MicroRNAs in cancer and PI3K‐Akt signalling pathway, which revealed these pathways might be related to the BTCC occurrence.

Afterwards, we parallelly performed WGCNA with those 2114 DEmRNAs to screen co‐expression genes modules relevant to clinical traits, especially tumour stage. Ultimately, the royal blue module with 11 genes was most significantly associated with tumour stage (BNC2, COL10A1, COL11A1, HEPH, KIF26B, METTL11B, MMP11, OMD, PLPP4, SGCD and VSTM4). By conducting survival analyses for them, we found they also possessed significance in the patients' outcome, which further revealed their potential relevance to BTCC occurrence, progression and prognosis.

Due to no common genes between ceRNA network and these 11 genes, we stepped back and extracted genes in all modules with the highest correlation coefficient. With a cut‐off |Cor| > 0.1 and *p* < 0.01, 100 genes were selected out. Predominant biological process of them included following GO terms: negative regulation of cell migration and muscle contraction, extracellular matrix organization. In addition, they participated in seven KEGG pathways: Vascular smooth muscle contraction, dilated cardiomyopathy, cGMP‐PKG signalling pathway, microRNAs in cancer, calcium signalling pathway, hypertrophic cardiomyopathy (HCM) and ECMreceptor interaction.

Totally, nine genes were both from ceRNA network and corelated with BTCC stage, including ZFPM2, AKAP12, ZEB1, RECK, THBS1, CYBRD1, JAZF1, FBXL7 and ZCCHC24. The ceRNA network contained them was composed another 13 miRNAs and 70 lncRNAs. Depending on the massive survival analysis used R package ‘survival’ and ‘*q*‐value’, seven mRNAs, three RNAs and eight lncRNAs were showed significance with BTCC overall survival (OS). Additionally, The RNAs in AC112721.1/LINC00473/AC128709.1‐hsa‐mir‐195‐ RECK and LINC00460‐hsa‐mir‐429‐ZFPM2 axes were simultaneously corelated with OS, which might indicate subsequent experimental direction. Except for AC112721.1 and AC128709.1, other lncRNAs, miRNAs and genes have been researched of their role in some types of tumours. For example, researchers demonstrated that LINC00473 antagonized miR‐195 to regulate the pathogenesis of Wilms tumour via IKKα.^33^ There were other scholars validated that the miR‐429 determined poor outcome and inhibited pancreatic cancer through targeting TBK1.^34^ Walsh and his colleagues found that RECK can participate in metastasis of breast carcinoma via regulation of STAT3‐dependent switch.^35^ However, functions of these genes in BTCC have not been fully elucidated and worth to be explored. As researchers reported that AKAP12 serves as a tumour suppressor in various cancers such as prostate, breast and ovarian cancers.^36^, ^37^, ^38^ AKAP12 acts as a regulator of mitogenesis by anchoring key signal proteins such as PKA, PKC and cyclins. Here, the GSEA results also showed that G2M checkpoint pathway was significantly enriched in AKAP12 low expression group, indicating cell cycle regulation role of AKAP12 in progression of bladder cancer. Moreover, in the RT‐PCR experiment, we revealed that AKAP12 was downregulated in BTCC tissues. Our studies suggest that AKAP12 is a candidate gene that could provide new strategies for finding biological markers for BTCC.

More and more mechanisms were identified functioning in tumours with the rapid experimental revolution and promising to be potential therapy targets. In addition, various coding and noncoding RNAs have become biomarkers for tumour screening and diagnosis, and prediction of progression or survival. Our study shed light on new clues of hub genes in BTCC using competing endogenous RNA network construction integrated with WGCNA. However, several limitations exert in this study. Firstly, this study was conveyed mainly based on the data from BLCA‐TCGA cohort; further validation in vitro and in vivo was indispensable and remained to be conducted. Secondly, expression validation cohort was relatively small; furthermore, samples should be collected to validate the expression of candidate biomarkers through western blot and Immunohistochemistry assay.

## 5 CONCLUSION

Taken together, we structured a ceRNA network with 83 DElncRNAs, 25 DEmiRNAs and 67 DEmRNAs with TCGA BTCC datasets. By WGCNA, the hub gene co‐expression module with 11 genes was found corelated with BTCC tumour stage and prognosis after survival analyses. Ultimately, we put 100 highly stage‐related genes into the above constructed ceRNA network and then constructed another new network. Among them, all elements in AC112721.1/LINC00473/AC128709.1‐hsa‐mir‐ 195‐RECK and LINC00460‐hsa‐mir‐429‐ZFPM2 axes were simultaneously corelated with OS. AKAP12 is a candidate gene that have directive meanings for further experimental study on BTCC.

## AUTHOR CONTRIBUTIONS

**Hai Wang:** Conceptualization (equal); resources (equal). **Lei Gao:** Data curation (equal); formal analysis (equal). **Yin Chen:** Data curation (equal); methodology (equal). **Lei Zhang:** Conceptualization (equal); resources (equal); writing – original draft (equal). **Yu Bai:** Data curation (equal); formal analysis (equal). **Cuiping Zhao:** Data curation (equal); project administration (equal); resources (equal). **Lifeng Zhang:** Conceptualization (equal); data curation (equal). **Li Zuo:** Conceptualization (equal); project administration (equal). **Heyun Sun:** Writing – review and editing (equal).

## FUNDING INFORMATION

This work is supported by Changzhou High‐Level Medical Talents Training Project (2022CZBJ057). Jiangsu Province ‘333’ project. Project of Changzhou Municipal Health Commission Science and Technology (No. WZ201917), Jiangsu Provincial Health Commission project (BJ21011).

## CONFLICT OF INTEREST STATEMENT

None declared.

## Supporting information


Table S1

Table S2

Table S3

## Data Availability

All data from the current study could be acquired from the authors after a reasonable request.
